# The non-coding transcriptome as a dynamic regulator of cancer metastasis

**DOI:** 10.1007/s10555-013-9455-3

**Published:** 2013-12-18

**Authors:** Francesco Crea, Pier Luc Clermont, Abhijit Parolia, Yuzhuo Wang, Cheryl D. Helgason

**Affiliations:** 1 Experimental Therapeutics, BC Cancer Research Centre, Vancouver, BC Canada; 2 Department of Surgery, University of British Columbia, Vancouver, BC Canada; 3 Interdisciplinary Oncology Program, Faculty of Medicine, University of British Columbia, Vancouver, BC Canada; 4 Honours Biotechnology, Department of Microbiology and Immunology, University of British Columbia, Vancouver, BC Canada; 5 The Vancouver prostate Centre, Vancouver General Hospital, Vancouver, BC Canada; 6 Experimental Therapeutics, B.C. Cancer Agency, 675 West 10th Avenue, Vancouver, BC V5Z 1L3 Canada

**Keywords:** Long non-coding RNAs, Small nucleolar RNAs, Non-coding interactome, Metastasis, Epigenetics

## Abstract

Since the discovery of microRNAs, non-coding RNAs (NC-RNAs) have increasingly attracted the attention of cancer investigators. Two classes of NC-RNAs are emerging as putative metastasis-related genes: long non-coding RNAs (lncRNAs) and small nucleolar RNAs (snoRNAs). LncRNAs orchestrate metastatic progression through several mechanisms, including the interaction with epigenetic effectors, splicing control and generation of microRNA-like molecules. In contrast, snoRNAs have been long considered “housekeeping” genes with no relevant function in cancer. However, recent evidence challenges this assumption, indicating that some snoRNAs are deregulated in cancer cells and may play a specific role in metastasis. Interestingly, snoRNAs and lncRNAs share several mechanisms of action, and might synergize with protein-coding genes to generate a specific cellular phenotype. This evidence suggests that the current paradigm of metastatic progression is incomplete. We propose that NC-RNAs are organized in complex interactive networks which orchestrate cellular phenotypic plasticity. Since plasticity is critical for cancer cell metastasis, we suggest that a molecular interactome composed by both NC-RNAs and proteins orchestrates cancer metastasis. Interestingly, expression of lncRNAs and snoRNAs can be detected in biological fluids, making them potentially useful biomarkers. NC-RNA expression profiles in human neoplasms have been associated with patients’ prognosis. SnoRNA and lncRNA silencing in pre-clinical models leads to cancer cell death and/or metastasis prevention, suggesting they can be investigated as novel therapeutic targets. Based on the literature to date, we critically discuss how the NC-RNA interactome can be explored and manipulated to generate more effective diagnostic, prognostic, and therapeutic strategies for metastatic neoplasms.

## Introduction

For many years, the so-called central dogma of molecular biology has been dominant in cancer research, as well as in all other bio-medical fields [1]. This dogma assumes that the information flow in a cell is uniquely directed from DNA to RNA to protein, and that proteins are ultimately responsible for cell phenotype. This paradigm has been challenged by several new discoveries, with perhaps the most important being the observation that while 90 % of the genome is transcribed, just 2 % of the RNA is translated into proteins [2, 3]. Non-coding RNAs include the well-characterized ribosomal-RNAs (rRNAs) and transfer-RNAs, as well as micro-RNAs, which have been demonstrated as useful biomarkers and therapeutic targets in several neoplasms [4–6]. Additionally, long non-coding RNAs (lncRNAs) and small nucleolar RNAs (snoRNAs), two less investigated classes of non-coding RNAs, are emerging as unexpected determinants of cancer initiation, progression, and metastasis.

LncRNAs are arbitrarily defined as RNA sequences longer than 200 bp with no protein-coding function [7]. This wide definition includes pseudogenes, micro-RNA precursors as well as RNAs interacting with epigenetic effectors and splicing factors. This multi-faceted group of genes is transcribed from as many as 10,000 different genetic *loci* in the human genome [8] and is involved in the regulation of many physiological and pathological processes [9]. Although only a small percentage of human lncRNAs have been characterized so far, notably most of them display either oncogenic or tumor-suppressive functions [10].

SnoRNAs are another abundant class of non-coding RNAs, encoded by approximately 500 different *loci* in the human genome. These sequences are 60–300 base pairs long and have been classically associated with “housekeeping” functions, like rRNA modification and splicing [11, 12]. Due to their supposedly stable expression, some snoRNAs have even been used as reference genes in cancer studies on microRNAs [13]. However, emerging evidence indicates that snoRNAs can play several non-classical roles, including reactive oxygen species scavenging in the cytoplasm and being precursors for microRNA-like molecules [14–16]. It is likely that the whole spectrum of snoRNA functions has not yet been fully elucidated. In parallel with those novel molecular mechanisms, it has become apparent that snoRNAs might play a role in cancer progression [17].

Metastatic spreading is the main cause of cancer-related deaths [18]. It is well known that this process requires that cancer cells display an abnormal phenotypic plasticity, allowing them to undergo a defined number of phenotypic alterations (e.g., epithelial-to-mesenchymal transition, tissue invasion, anchorage-independent growth, and homing to distant tissues) [19]. Despite a deep characterization of the protein-related pathways involved in each step [20], we are not currently able to account for all the molecular mechanisms orchestrating this complex phenomenon. In this paper, we summarize the emerging evidence showing that lncRNAs and snoRNAs could play crucial roles in several steps of the metastatic progression. We also propose a model suggesting how non-coding RNAs and epigenetic effectors can synergize to shape cancer cell plasticity and drive the colonization of distant organs. Finally, we indicate how fundamental research on uncharted genomic regions could reshape the classical landscape of translational research and eventually improve the clinical outcome of cancer patients.

## Long non-coding RNAs

Recent transcriptomic analyses of human neoplasms have challenged the common belief that random sequential genetic mutations occurring in protein-coding genes underlie the acquisition of metastatic phenotype [21, 22]. Cancer metastasis is a multi-step process that requires dynamic transcriptional and translational regulation over time in response to distinct selective pressures conferred by an evolving extracellular environment [23–25]. Consequently, such a complex series of events imply that additional factors must synergize with genetic alterations to induce cancer spread [26, 27]. In line with this idea, an increasing number of studies report that lncRNAs represent some of the most differentially expressed transcripts between primary and metastatic cancers [28, 29]. While some investigators have proposed that lncRNAs mainly represent the product of leaky transcription [2], there is now considerable evidence indicating that deregulation of these molecules functionally drives physiological and pathological processes. As genetic mapping and functional characterization of lncRNAs proceeds, electronic databases have been created to catalogue this mounting information [30] and link it to human diseases [31].

The emergence of lncRNAs in cancer biology is already revolutionizing our approach to translational oncology. The past decade has revealed several examples of differentially expressed lncRNAs carrying diagnostic and/or prognostic value, some of which are now routinely used in the clinic [32]. Interestingly, many lncRNAs are consistently associated with clinical parameters indicative of metastasis in a wide spectrum of tumor types [33, 34]. A notorious example of such an oncogenic lncRNA is metastasis-associated lung adenocarcinoma transcript 1 (MALAT1) which, as indicated by its name, was initially found over-expressed in lung cancer metastases [35]. Studies in different neoplasms have linked higher MALAT1 expression with shorter metastasis-free survival (MFS) [35], deeper tissue invasion [36], higher histological grade [37], and shorter overall survival (OS) [38]. Analogous to MALAT1, Homeobox transcript antisense RNA (HOTAIR) represents another lncRNA strongly associated with metastatic progression. Over-expression of HOTAIR occurs in about 30 % of breast neoplasms and significantly predicts shorter MFS and OS independently of tumor size, stage, and hormone receptor status [39]. Studies in other cancer types also identified positive correlations between HOTAIR expression and lymph node metastasis [40], lymph-vascular invasion [41], as well as shorter recurrence-free survival [42]. While representing only a few examples of an increasing body of literature, MALAT1 and HOTAIR provide a solid rationale for developing more lncRNA-based tests aimed at assessing the pro-metastatic potential of primary tumors.

While previously discussed lncRNAs promote metastatic spreading, there are also many examples of oncosuppressive lncRNAs whose down-regulation strikingly associates with metastatic behavior in the clinical setting. Among them, BM742401 exhibits decreased expression in more aggressive cancers, correlating with metastatic properties and decreased survival in gastric cancer tissues [43]. Moreover, ectopic expression of BM742401 significantly decreases cellular invasion and migration in part by modulating the activity of matrix metalloproteinases. Another interesting finding is that the large majority of cancer-related single-nucleotide polymorphisms (SNPs) are found in non-coding regions. In fact, only 3.3 % of cancer-related SNPs ultimately lead to a change in amino acid at the protein level, while more than 80 % of SNPs map to expressed non-coding regions [44]. Some of these SNPs have been directly linked to cancer metastasis. One such example is a SNP found in the CCAT2 lncRNA (rs6983267), mapping to 8q24 [45, 46]. In inflammatory breast cancer, this SNP is associated with the occurrence of metastasis and independently predicts outcome [47]. Another study showed that CCAT2 increases cell invasion and motility *in vitro* and *in vivo*, correlating with shorter MFS in colon and breast cancer [46]. Even if the majority of data on lncRNA in cancer are just correlative, their abundance suggests that these molecules play much more than a passive role in cancer metastasis. In the following sections, we will review known mechanisms of lncRNA-dependent cancer progression, which can be primarily divided into nuclear and extra-nuclear actions.

### Nuclear mechanisms of lncRNA-induced cancer progression

**Fig. 1:**
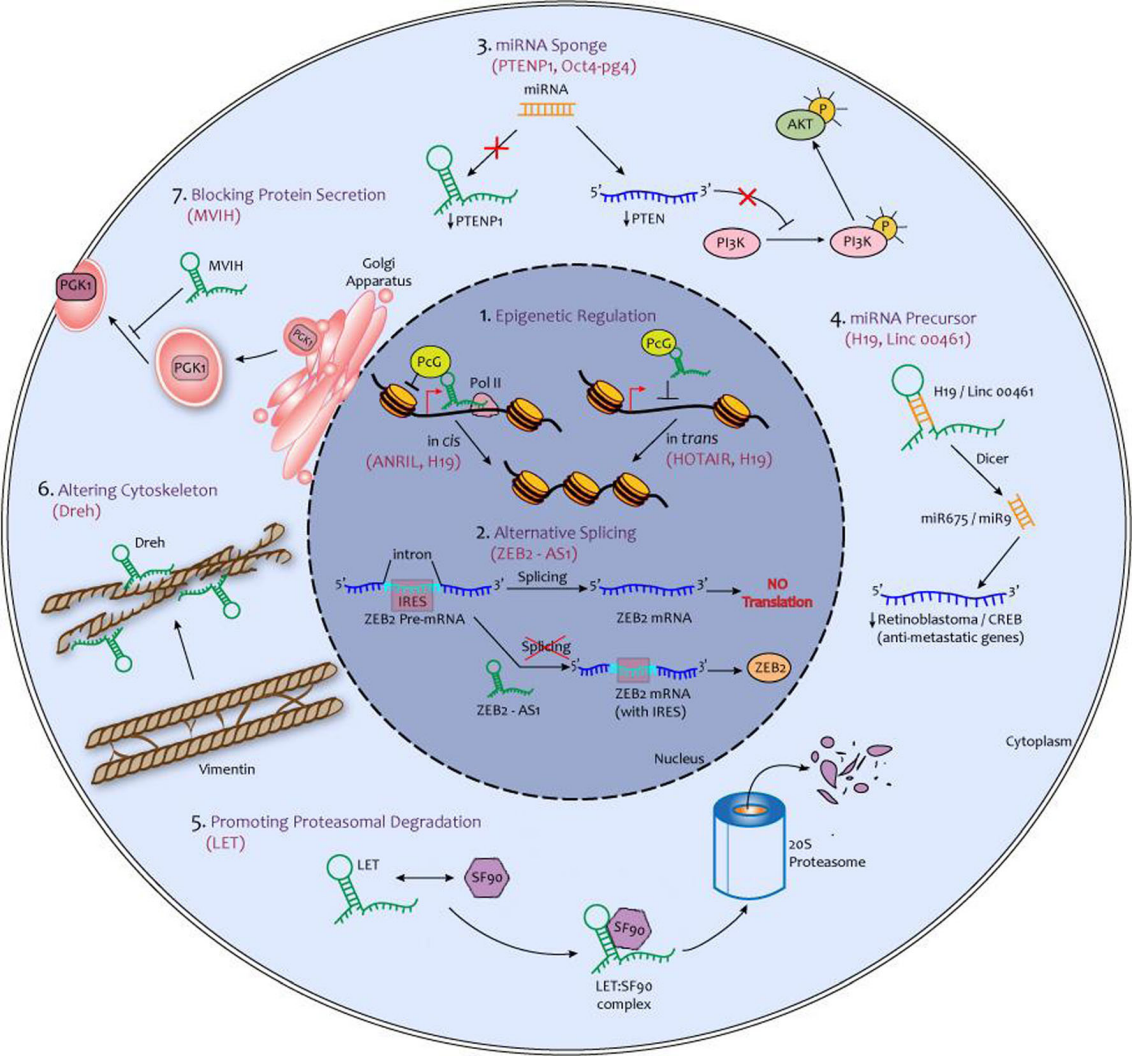
Reported mechanisms by which lncRNAs can promote cancer metastasis. *1* to *7* illustrate various mechanisms by which long non-coding RNAs (lncRNAs) may contribute to attainment of the metastatic phenotype in cancer cells. Mechanisms *1* and *2* are nuclear, while mechanisms *3*–*7* are extra-nuclear. The name of one or two lncRNAs functioning through the specified mechanism is shown in *brackets* (e.g., ZEB2-AS1 controls alternative splicing)

The heavily altered lncRNA transcriptome in human cancer metastases has opened the way for a number of functional studies to uncover the molecular mechanisms by which lncRNAs influence cancer progression (summarized in Fig. 1). It is now well accepted that lncRNAs are versatile macromolecules with the potential to play multiple roles at different stages of the metastatic process [48, 49]. While lncRNAs vary extensively in structure and activity, their dominating function occurs by physically interacting with epigenetic complexes and recruiting them to specific *loci* either in *cis* or in *trans* [50]. By doing so, lncRNAs can dynamically orchestrate large-scale transcriptional programs required to spatially and temporally guide the cell throughout the different stages of the metastatic process [39, 51]. An emerging theme for these lncRNA lies in their ability to cooperate with polycomb repressive complexes (PRC1 and PRC2), which have been notoriously associated with metastatic progression and phenotypic plasticity (Fig. 1, mechanism 1) [52–55]. In fact, it is estimated that about 20 % of all intergenic lncRNAs have the ability to bind PRC2 [56], and a very large number of them also regulate the activity of PRC1 [57–59]. These findings indicate that lncRNAs likely play an important functional role in epigenetic regulation of metastasis, notably through interactions with PcG proteins.

Adding to the complex interplay between epigenetics and lncRNAs, some genes encoding lncRNAs may also undergo genomic imprinting [60]. The first lncRNA ever described, H19, is the classical example of such gene [61, 62]. H19 is a paternally imprinted lncRNA antisense to the insulin-like growth factor 2 (IGF2) *locus* which exhibits loss of imprinting and very strong up-regulation in a wide range of metastatic neoplasms [63–65]. Supporting a driving role for H19 in metastasis, both silencing and over-expression of H19 was shown to modulate metastatic behavior in bladder cancer [51]. A multi-cancer study also found that H19 expression was significantly higher in liver metastases compared to the primary tumors from which they originated [66]. The H19 promoter is known to be negatively regulated by p53 [67] and positively regulated by c-Myc [68], E2F [69], and HIF1-α [67], providing a partial explanation for its high expression in human metastases. To illustrate the extent of H19 over-expression in aggressive tumors, gene therapy strategies aimed to drive diphtheria toxin A expression under the control of the H19 promoter are currently being tested clinically for bladder, ovarian, and pancreatic, and colorectal cancer metastases [70–73].

Studies aimed to uncover the mechanistic basis by which H19 promotes metastatic progression have interestingly shown that H19 can play multiple molecular functions [74], some of which are dependent on the particular splicing variant [75]. In bladder cancer, H19 directly binds EZH2 and recruits PRC2 to the E-cadherin promoter, thereby suppressing E-cadherin expression and promoting epithelial-to-mesenchymal transition (EMT) [76]. In addition, H19 can also regulate gene expression post-transcriptionally by serving as a precursor for the miRNA-675, which directly targets the tumor suppressor Retinoblastoma (Rb) [77, 78]. In colon cancer cells, up-regulation of miRNA-675 decreases Rb levels, which subsequently increases colony-formation ability in soft agar, a phenotype associated with acquisition of anchorage-independent growth [78]. Based on this property, we propose that this miRNA-associated feature of H19 may therefore enhance the survival of circulating tumor cells. All these data highlight the diverse functional nature of H19 resulting in its potential to actively regulate multiple stages of metastasis.

Recently, another chromatin-associated lncRNA, HOTAIR, has attracted investigators’ attention due to its profound pro-metastatic properties. HOTAIR has served as a paradigm for epigenetic regulation by lncRNAs through its ability to serve as a molecular scaffold for two histone-modifying complexes that promote heterochromatin formation in *trans* [39]. Mechanistically, HOTAIR can simultaneously interact with both PRC2 and LSD1/CO-REST, which catalyze histone H3K27 trimethylation and H3K4 demethylation, respectively. When over-expressed, HOTAIR targets these repressive complexes to anti-metastatic *loci* and induces an epigenetic reprogramming enhancing cellular plasticity [39, 41]. Moreover, there are reports suggesting HOTAIR might also control DNA methylation, as its depletion in Hep-2 cells lead to a decrease in phosphatase and tensin homolog (PTEN) promoter methylation [79]. These findings highlight HOTAIR’s versatility and support the idea that lncRNAs can act as molecular scaffolds to bridge multiple epigenetic complexes and therefore regulate large-scale chromatin dynamics.

While the previously discussed lncRNAs acted mostly in *trans*, there are also a number of these transcripts that regulate chromatin dynamics in *cis* (Fig. 1, mechanism 1) [80]. Antisense non-coding RNA in the INK4 locus (ANRIL) is one characterized lncRNA working in this fashion [81]. ANRIL negatively regulates expression of the CDKN2A locus, which encodes three proteins known to inhibit tumor progression: p14^ARF^, p15^INK4B^, and p16^INK4A^ [59]. This mechanism first involves a direct binding between ANRIL and SUZ12, which recruits PRC2 and activates its catalytic activity at the CDKN2A *locus* [82]. Following H3K27 trimethylation by PRC2, ANRIL then serves as a tether to recruit the PRC1 subunit chromobox homolog 7 (CBX7), which further represses transcription [57]. CBX7 is well known for regulating cellular lifespan through bypassing replicative senescence and providing unlimited self-renewal ability, and does so through the repression of the CDKN2A locus [83–85]. Since metastatic cells that have reached a new organ must repopulate a whole tumor, it is implied that they must have acquired unlimited replicative ability despite receiving senescence signals [86, 87]. Increased ANRIL expression coupled with PcG activity at the CDKN2A locus therefore represents one way by which cells can achieve unlimited self-renewal ability and therefore improve the efficacy of metastatic tumor growth.

While most nuclear lncRNAs control multiple cellular processes through epigenetic mechanisms, a number of them also exert their function post-transcriptionally by regulating alternative splicing (Fig. 1, mechanism 2) [88]. Recent studies have demonstrated that the Zeb2 antisense RNA 1 (ZEB2-AS1) promotes tumor invasion by directly linking the processes of alternative splicing and metastasis, more specifically by inducing EMT [89]. ZEB2-AS1 overlaps with an intron in the 5′ end of the ZEB2 gene which contains an internal ribosomal entry site (IRES) required for its translation [90]. However, this 5′ intron can be spliced, which abrogates the translation of Zeb2 mRNA into protein. Upon transcriptional induction by Snail, ZEB2-AS1 is expressed and prevents the splicing of the Zeb2 5′-untranslated region. This allows retention of the ZEB2 I.E. and therefore allows synthesis of Zeb2 protein [89]. Since Zeb2 can directly inhibit E-cadherin expression and activate the EMT program, ZEB2-AS1 expression favors tumor invasion [91, 92]. This splicing mechanism is consistent with the finding that Zeb2 protein levels increase in cells undergoing EMT while Zeb2 mRNA levels remain unchanged [89]. In addition to ZEB2-AS1, MALAT1 is another nuclear-retained lncRNA which has been directly linked to cancer metastasis and interestingly also plays a role in alternative splicing. Also called nuclear-enriched abundant transcript 2, MALAT1 localizes to nuclear speckles where it regulates the phosphorylation of the pre-mRNA splicing factors such as SR proteins, thereby controlling their localization and activity [93, 94]. However, while MALAT1 has been directly linked to splicing control in some cellular contexts [95], the molecular mechanisms by which this lncRNA promotes metastasis need to be fully dissected [96]. Nonetheless, by regulating SR protein activity, MALAT1 has the potential to actively promote tumor dissemination by modifying the splicing patterns of metastasis-related transcripts.

Besides a putative role in alternative splicing, MALAT1 may also contribute to cancer metastasis through other mechanisms. In fact, many studies have reported that MALAT1 plays an active role in controlling gene transcription. In lung adenocarcinoma, MALAT1 directly regulates the expression of pro-migratory genes such as CTHRC1, CCT4, HMMR, and ROD1 [97]. MALAT1 can also enhance the metastatic potential of bladder cancer by modulating the expression of genes involved in EMT [98]. Another interesting study revealed that MALAT1 can interact with the protein CBX4, a member of the pro-metastatic complex PRC1 [99], and regulates its subnuclear shuttling between polycomb bodies and interchromatin granules [58]. Additionally, MALAT1 may also contribute to metastasis by generating a short tRNA-like molecule (called mascRNA) upon ribonuclease P activity [100]. While this phenomenon has been well-described, the function of the resulting mascRNA remains unclear. Future studies may reveal similar post-transcriptional processing mechanisms altering localization and function of specific non-coding RNAs, adding another layer of complexity to the metastasis-driving lncRNA transcriptome.

### Extra-nuclear mechanisms of lncRNA-induced cancer progression

One of the most interesting discoveries in recent years has been that not only can lncRNAs be actively shuttled in the cytoplasm, they can also carry out multiple pro-metastatic functions in this subcellular compartment (Fig. 1, mechanisms 3–7) [101, 102]. For example, a subclass of lncRNAs called pseudogenes or competing endogenous RNAs (ceRNAs) can alter gene expression post-transcriptionally by acting as miRNA buffers (Fig. 1, mechanism 3). Pseudogenes are thought to arise from the duplication of protein-coding genes followed by genomic alterations that abolish their ability to be translated [103]. Hence, pseudogenes may retain functional miRNA-binding sites [104], thereby buffering the pool of miRNAs targeting their protein-coding counterpart [105]. This phenomenon confers capital importance to pseudogenes related to metastasis-driving genes. One of the ceRNA most relevant to cancer spreading is PTENP1, a tumor suppressive pseudogene related to PTEN phosphatase [106]. Genomic loss and down-regulation of PTENP1 is often found in aggressive human cancers, an event correlating with PTEN down-regulation through a miRNA-dependent mechanism [107, 108]. Loss of PTEN function leads to PI3K phosphorylation and activation of the Akt pathway, which subsequently drives cancer cell invasion and migration [109–111]. Interestingly, a PTENP1 antisense (PTENP1-AS) transcript was subsequently identified. PTENP1-AS inhibits PTENP1 expression through *cis* (RNA-RNA binding) and *trans* (PRC2-dependent silencing) mechanisms, thereby playing an oncogenic role. This multi-layer regulatory network occurring in one *locus* suggests that ceRNAs may control multiple targets in cancer cells, thereby acting either as pro- or anti-metastatic genes in a context-specific manner [46]. As previously discussed for H19, many lncRNAs can themselves serve as precursors for miRNAs, some of which are known to play important roles in cancer progression (Fig. 1, mechanism 4) [77]. Another example is linc00461 which is a precursor for miRNA-9 that has the potential to target cAMP response element-binding protein (CREB) [112]. It has been shown that miRNA-9 over-expression inhibits proliferation but promotes migration of glioma cells through CREB repression. Interestingly, CREB and miRNA-9 are involved in an auto-regulatory feedback loop since CREB itself can inhibit miRNA-9 expression, suggesting interplay between different types of non-coding RNAs may regulate cancer metastasis [112, 113]. With so many lncRNAs being transcribed in the human genome [8], it is likely that many other metastasis-related miRNAs derived from lncRNAs will be discovered.

Beside miRNA regulation, extranuclear lncRNAs may also promote cancer progression through specific interactions with cytoplasmic proteins. The nature and outcome of these interactions can be highly diverse, thereby allowing multiple molecular processes to be regulated by lncRNAs. For example, one recent study has shown that the lncRNA low expression in tumor (lncRNA-LET) can inhibit metastasis by physically associating with NF90 and promoting its proteasomal degradation (Fig. 1, mechanism 5) [114]. NF90 is an oncogenic RNA-binding protein known to stabilize HIF1-α transcripts, thereby promoting hypoxia-mediated metastasis [115, 116]. In hepatocellular carcinomas, colorectal cancers, and squamous cell lung carcinomas, lncRNA-LET is significantly down-regulated which leads to NF90 up-regulation, HIF1-α mRNA stabilization, and subsequent increase in tumor cell invasiveness under hypoxic conditions [114]. While an influence on proteasomal degradation has only been proposed recently, we believe a thorough analysis of the dynamic lncRNA-protein interactome may reveal similar molecular mechanisms promoting cellular plasticity.

Another recently discovered mechanism by which lncRNA can promote metastasis is by binding specific cytoskeletal proteins (Fig. 1, mechanism 6) and directly altering their 3D structure and function [117]. Through this action, it is thought that cancer cells can dynamically regulate their motility in response to changes in lncRNA expression. Such a mechanism occurs in hepatocellular carcinoma (HCC) cells expressing the hepatitis B virus X protein. These cells express a lncRNA called Dreh which localizes to the cytoplasm and physically interacts with the intermediate filament (IF) protein vimentin, a marker of mesenchymal differentiation and EMT [118]. Binding of Dreh alters vimentin’s structure and consequently inhibits its EMT-promoting function [117]. Consistent with this mechanism, Dreh over-expression was shown to significantly inhibit the metastasis of HCC cells [117]. Since the cytoskeleton plays such a crucial role in controlling cell motility [119], altering its function through lncRNA binding may emerge as a frequent molecular event underlying cancer metastasis.

Finally, there is evidence supporting the idea that lncRNAs may dynamically control the composition of the tumor microenvironment by altering the secretion of metastasis-regulating proteins (Fig. 1, mechanism 7). A recent study demonstrated that the lncRNA associated with microvascular invasion in hepatocellular carcinoma (lncRNA-MVIH) directly inhibits secretion of phosphoglycerate kinase 1 (PGK1) [120]. Since PGK1 promotes metastasis in some cancer types [121], blocking its extracellular release could enhance cancer dissemination. This process has also been closely linked with an increase in pro-angiogenic potential of the tumor stroma, highlighting the tight relationship between angiogenesis and metastasis [122]. While the exact mechanism through which lncRNA-MVIH inhibits PGK1 secretion remains elusive, a dynamic influence of lncRNAs on the extracellular milieu is consistent with the idea that they represent master regulators of cancer metastasis. These results highlight once again the diverse nature of lncRNA interactions and strengthen the rationale for the use of more lncRNA-based tools in translational oncology.

In summary, the considerable number of differentially expressed lncRNAs in metastatic tumors, coupled with the diversity of their molecular functions, challenges the assumption that phenotypic plasticity is conferred solely by alterations in protein-coding genes. The number of publications linking lncRNA and metastasis has been increasing exponentially in the past decade, and there is good reason to believe that this trend will continue in the years to come.

## Small nucleolar RNAs

**Fig. 2:**
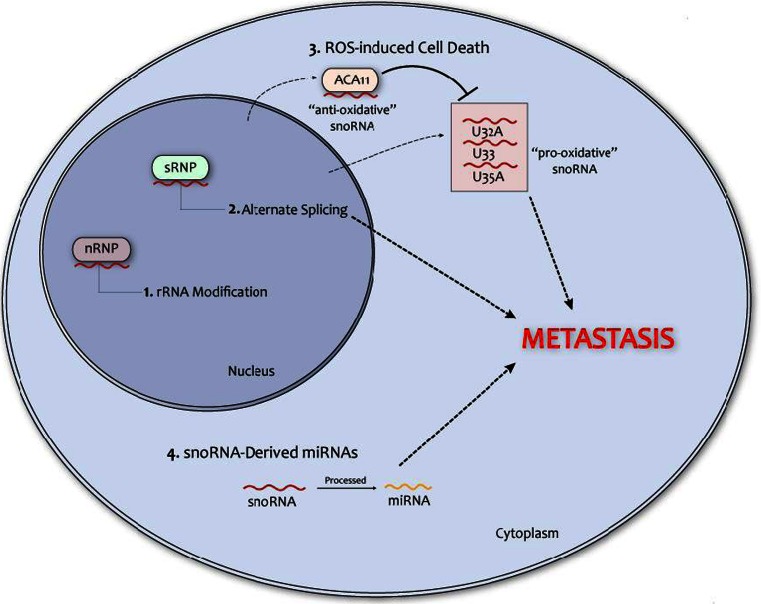
Putative mechanisms through which snoRNAs can promote cancer metastasis. *1* Depicts the classical role of small nucleolar RNAs (snoRNAs) regulating rRNA modification, while *2* to *4* depict the non-classical roles through which snoRNAs may trigger acquisition of the metastatic phenotype in cancer cells. Mechanisms *1* and *2* are nuclear, while *3* and *4* are extra-nuclear. *Dotted lines* indicate that the relationship to metastasis is hypothetical

SnoRNAs are an evolutionary conserved class of RNAs expressed by all eukaryotic cells [123]. They were first discovered and named after their localization in the nucleolus, where they contribute to ribosomal RNA (rRNA) biogenesis [124]. The synthesis of rRNA in the nucleolus requires an intricate sequence of post-transcriptional modifications [125]. SnoRNAs are recruited into multi-protein complexes to form ribonucleoproteins (RNP). Each snoRNA anneals with a complementary rRNA sequence, thereby guiding the catalytic activity of the nucleolar RNP (nRNP) to a specific site (Fig. 2). Two main snoRNA (and RNP) categories are known: C/D box, which catalyze 2′-O-methylation; H/ACA-box, which catalyze pseudouridylation [123, 125]. Both modifications are required for accurate rRNA-mediated protein biogenesis [125]. SnoRNAs display considerable genetic redundancy as the human genome contains at least 500 distinct *loci*, while encoding for approximately 200 functional snoRNAs [126]. Indeed, multiple copies of the same snoRNA are encoded by different *loci*, almost all of which are located in introns of protein-coding genes. Notably, bioinformatic analysis revealed the presence of an even larger number of supposedly non-functional snoRNA *loci* (e.g., pseudogenes) in the human genome, raising the number of snoRNA-like genes to over 1,000 [127]. Since snoRNA functions are not completely elucidated, it is intriguing to speculate that snoRNA pseudogenes can play functional roles in mammalian cells, not unlike the pseudogene fraction of lncRNAs.

As research on snoRNAs progressed, it became apparent that these non-coding RNAs can have several non-classical functions. Recent evidence indicates that snoRNAs can be processed to obtain shorter sequences that are involved in the control of mRNA alternative splicing [128]. For example, SNORD115, which is selectively expressed in the brain, is processed into shorter sequences lacking the stem sequence that is crucial for the assembly into a functional nRNP, but still containing the antisense box needed for targeting complementary RNA [129]. These processed snoRNAs are assembled into nuclear RNPs involved in splicing control (sRNP). One of those sRNP-related snoRNAs is SNORD115, which targets the serotonin receptor 5-HT2C mRNA and inhibits its splicing. This mechanism is deregulated in Prader-Willi syndrome, a neural disease that is the most common genetic cause of obesity [130]. Bioinformatic and functional analysis revealed that SNORD115 controls the splicing of at least five more genes, two of which (TAF1 and PBRM1) have been implicated in the progression from localized to metastatic castration-resistant prostate cancer [129, 131, 132]. To date, there is not a direct molecular link between SNORD115 and prostate cancer. However, this data, coupled with the function of some oncogenic lncRNAs (e.g., ZEB2-AS1, MALAT1), strongly suggest that splicing control can be a mechanism by which some snoRNAs regulate cancer metastasis. SnoRNA molecular functions were further broadened by the discovery that snoRNA45 is processed to produce miRNA-like molecules in a Dicer-dependent, Drosha-independent manner [133]. This snoRNA-derived miRNA is processed in the cytoplasm and triggers post-translational gene silencing on complementary mRNAs. A subsequent study showed that at least 11 more snoRNAs can generate functionally active miRNAs [15]. Another group of SNORNAs (U32a, U33, and U35a) is able to shuttle to the cytoplasm and trigger cell death in response to oxidative stress [134]. Even if the authors did not manage to dissect the exact molecular mechanisms, this further cytoplasmic function of snoRNAs suggests that their roles might extend far beyond rRNA editing.

### Role of snoRNAs in cancer and metastasis

In parallel with the emergence of non-classical molecular functions, it has become apparent that snoRNAs might play a role in human diseases, including cancer [17, 135, 136]. Since evidence on the oncogenic roles of snoRNAs is less advanced compared to the lncRNA field, most studies to date have focused on the tumorigenic and proliferative functions of those genes, overlooking their putative role in metastatic spreading. Nonetheless, we think that this emerging evidence strongly suggests that snoRNAs are also involved in late stages of cancer progression. Due to their widespread requirement for proper protein synthesis, snoRNAs have long been thought to work as “housekeeping” genes in different cell types, and were therefore used as references in studies measuring the expression of miRNAs in cancer patients [137]. Gee and coworkers [13] challenged this assumption, finding that snoRNA expression in cancer samples is as variable as miRNA expression. In addition, they demonstrated that SNORD48 expression significantly decreases from low- to high-grade breast cancers, and that SNORD44 up-regulation is associated with better prognosis in both breast and non-small cell lung cancer. Even if no functional studies were performed, those results suggest that SNORD44 and 48 might play a tumor suppressive function in these neoplasms. In keeping with those results, Esteller and co-workers [138] identified three snoRNAs which are frequently silenced by DNA methylation in a panel of cancer cell lines and clinical samples. Notably, the same snoRNAs are not methylated and are normally expressed in non-neoplastic tissues. Since epigenetic silencing of tumor suppressor genes in a common feature of human neoplasms, these results suggest that at least some snoRNAs may function in tumorigenesis.

The first mechanistic insight on how snoRNAs can inhibit tumorigenesis came from a study aimed at identifying the genetic roots of a chromosomal deletion (6q14–q22) common to many cancer types [139]. To identify the tumor-suppressor gene located in this region, the authors narrowed the common region of deletion to a 2.5-Mb interval at 6q14–q15. Of the 11 genes located in this region and expressed in prostate tissues, only SNORD50 was mutated, demonstrated transcriptional down-regulation and inhibited colony formation in prostate cancer cells. A few years later, the same authors demonstrated that SNORD50 also plays a tumor-suppressive function in breast cancer [140]. Whether SNORD50 exerts its tumor-suppressive function through classical or non-classical mechanisms is still to be determined. In addition, the authors mainly showed that this gene is able to inhibit colony formation in cancer cells, a feature that is generally linked to the tumorigenic potential. Future studies should dissect the molecular pathways affected by this and other candidate tumor-suppressive snoRNAs. It would be particularly interesting to test the hypothesis that tumor-suppressive snoRNA play a role not only in tumorigenesis, but also in neoplastic progression, and particularly in metastasis.

A few snoRNAs have been also characterized as putative oncogenes. Among them is snoRNA42, whose *locus* is frequently amplified in lung cancer lesions. SnoRNA42 silencing leads to growth arrest and p53-dependent apoptosis in lung cancer cells [141]. Again, the exact molecular mechanism underlying this dramatic phenotypic change has not been revealed, but the authors proposed that a snoRNA42-derived miRNA could target p53, thereby inhibiting its pro-apoptotic function [17]. In multiple myeloma (MM) cells, ACA11 snoRNA is a crucial component of a RNP that silences riboproteins and snoRNAs implicated in the control of oxidative stress [142]. As a consequence, ACA11 enhances MM cell proliferation, inhibits oxidative stress response, and confers chemotherapy resistance.

The evidence presented above shows that snoRNAs can play tumor-suppressive or oncogenic functions in human neoplasms and that they can exert their action through several mechanisms, some of which probably remain to be discovered. Even if the link between snoRNAs and metastatic progression needs to be confirmed, we think that current knowledge suggests that this relationship should be investigated. Figure 2 shows at least three mechanisms by which oncogenic or tumor-suppressive snoRNAs can affect metastasis:

1. Splicing control. This is an important mechanism of action of ZEB2-AS1 and MALAT1 lncRNAs [89, 143], which exerts potent pro-metastatic function in several cancer cell types. Notably, many snoRNAs (e.g., SNORD115 [130]) exert similar splicing control on a wide range of genes, some of which have been implicated with prostate cancer progression to a metastatic stage [129, 131, 132]. It is therefore conceivable that, by controlling the splicing of metastasis-driving genes those snoRNAs could regulate the metastatic process.
2. Production of snoRNA-derived miRNAs. Classical miRNAs are master regulators of human cancer metastasis, by targeting multiple molecular pathways involved in each step of this complex phenomenon [144]. SnoRNA-derived miRNAs can play similar roles, thereby enhancing or inhibiting cancer progression.
3. Different sets of snoRNAs can enhance or inhibit cell death in response to oxidative stress [134, 142]. It has been recently shown that antioxidant pathways are activated by cancer cells to evade anoikis, a cell death program triggered by detachment from the extracellular matrix (ECM) [145]. The authors of this seminal paper showed that detachment from the ECM strongly increases the production of reactive oxygen species, which are toxic for most normal and cancer cells. Only neoplastic cells with the ability to activate antioxidant pathways can survive, thereby acquiring anchorage-independent growth and the ability to metastasize to distant organs. Based on this evidence, we propose that “anti-oxidative” snoRNAs (e.g., ACA11) could enhance, while “pro-oxidative” snoRNAs (e.g., U33) could inhibit, cancer cell anchorage-independent growth.

**Fig. 3:**
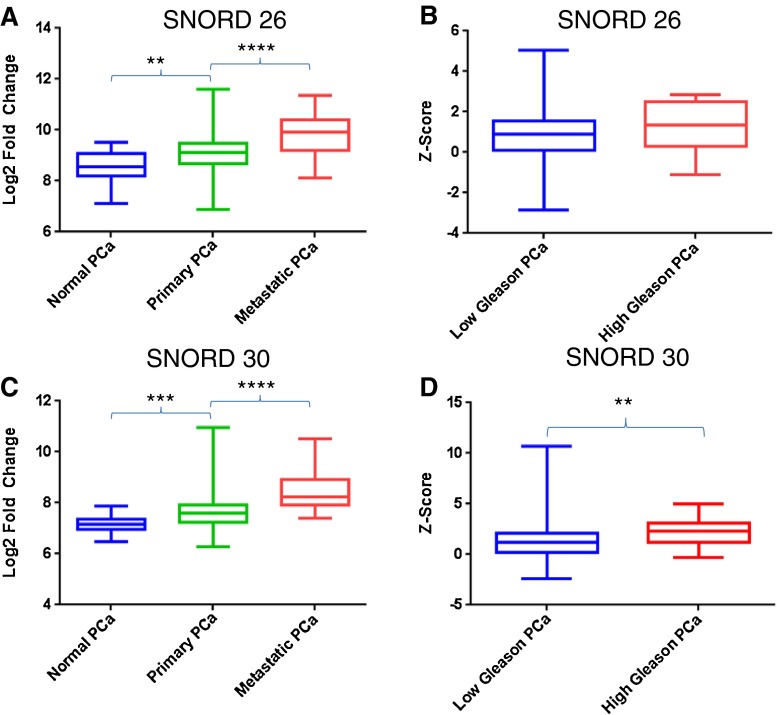
Expression of two snoRNAs in human prostate cancer. The analysis was performed using the MSKCC prostate adenocarcinoma database on cBioPortal [148]. A total of 267 different snoRNAs were ranked based on their ratios of average metastatic expression to average localized prostate cancer expression. Here we show the top two differentially expressed snoRNAs, SNORD26 and SNORD30. **a** and **c** Depict expression of SNORD 26 and SNORD 30, respectively, in normal individuals (29 samples) and patients with localized (131 samples) and metastatic (19 samples) prostate cancer. **p < 0.01, ***p < 0.001, and ****p < 0.0001 (one-way ANOVA and Tukey’s Test). **b** and **d** Show the expression of SNORD 26 and SNORD 30, respectively, in low gleason (117 samples) vs high gleason (22 samples) prostate cancer. *p< (unpaired, two-tailed student’s *T* test)

To date, we found only one study directly showing that snoRNAs are deregulated during metastatic progression. A deep sequencing analysis investigated the whole small RNA transcriptome in organ-confined *vs*. lymph-node-positive prostate cancer samples [123]. The authors found that the overall expression of miRNA decreased in higher tumor stages. Strikingly, the opposite occurred for snoRNAs, most of which were up-regulated in lymph-node-positive cases. These results are in keeping with the evidence that a generally increased snoRNA biogenesis is essential for breast cancer progression [146]. The authors of the deep sequencing analysis identified as many as 71 snoRNAs up-regulated (more than twofold) in higher stage PCa, but they subsequently focused only on the prognostic role of miRNA signatures. Thus, the clinical relevance of the deregulated snoRNA expression profile needs to be fully elucidated. We think that this early evidence provides the impetus for further analysis of snoRNA function in metastatic PCa. For this reason, we investigated snoRNA expression profiles in Cbio Cancer Genomic Portal (“Prostate Adenocarcinoma MSKCC 2010” dataset) a publically available gene expression database containing data from 29 normal prostatic tissues, 131 localized, and 19 metastatic PCa samples [147, 148]. We detected 267 snoRNA genes, and ranked them based on their differential expression in metastatic *vs*. primary prostate cancer. Figure 3 shows the two most differentially expressed snoRNAs, both showing a highly significant (*p* < 0.01, ANOVA and Tukey’s post-test) incremental increase in expression through progression from normal prostate to localized and then metastatic PCa samples. Notably, SNORD30 (which is also positively correlated with Gleason score, *p* = 0.01, unpaired two-tailed *T* test) was among the 71 differentially expressed snoRNAs described by Martens and co-workers [149].

Albeit preliminary and mainly based on gene expression patterns, these results provide compelling preliminary data suggesting that snoRNAs may be involved in metastatic progression, not unlike the better characterized miRNAs and lncRNAs. Future studies should investigate their role in other neoplasms, and more importantly the molecular mechanisms underlying their pro- or anti-metastatic functions. We believe that such analyses of this ancient class of non-coding RNAs have the potential to reveal unexpected molecular insights into the process of human cancer metastasis.

## Conclusion: the non-coding RNA interactome and its clinical implications

Despite being the major cause of cancer-related deaths, metastasis remains a poorly understood phenomenon [24]. This is partially due to the fact that metastatic dissemination is an extremely complex phenomenon requiring several molecular steps (e.g., invasion, migration, intravasation, and homing in distant tissues). It has been suggested that the phenotypic plasticity required for cancer cell dissemination might result from the interaction of different molecular networks involving protein-coding genes [20]. However, therapeutic strategies aimed at targeting some of these pathways have produced disappointing results in clinical trials [150–152]. We believe this discrepancy could be at least in part explained by the idea that protein-coding genes are not the driving factors in metastatic progression, and that instead they lie under the regulation of what we have designated the non-coding RNA interactome.

**Fig. 4:**
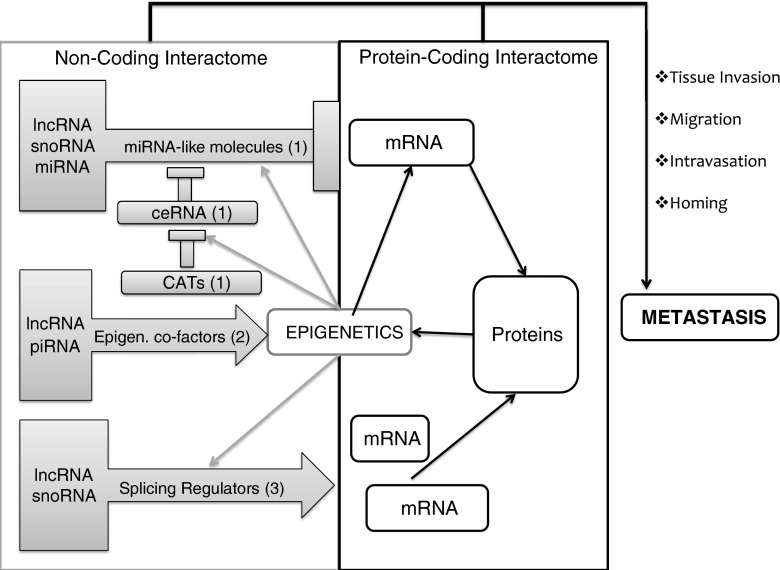
Molecular networks orchestrating cancer metastasis. The non-coding (*grey* boxes) and the protein-coding (white boxes) interactomes co-operate to drive each step of the metastatic cascade (e.g., tissue invasion, migration, intravasation, and homing in distant tissues). Different classes of NC-RNAs can play partially overlapping functions. We here summarize three main classes: *1* Antisense RNA inhibitors, including miRNA-like molecules, competitive endogenous RNAs (ceRNAs) and ceRNA-antisense transcripts (CATs); *2* epigenetic co-factors; *3* splicing regulators. Each component of the interactome can activate (*arrows*) or inhibit (*rectangles*) other components. Epigenetic gene regulation is depicted at the border of the two interactomes, since it can control the expression of both NC-RNAs and mRNAs. Moreover, epigenetic effectors are composed by both proteins and lncRNAs

The recent discovery that the non-coding transcriptome exceeds the protein-coding one by number and diversity, and that many NC-RNAs are involved in cancer progression, suggests that an uncharted molecular network might orchestrate cancer cell dissemination (Fig. 4). It is becoming increasingly clear that different classes of NC-RNAs are functionally interconnected, and that their coordinated interactions regulate cellular phenotypic changes in both physiological and pathological conditions [153]. Based on current data, we believe the proposed NC-RNA interactome exerts its pro-metastatic function mainly by dynamically orchestrating three fundamental processes: epigenetic gene regulation, alternative splicing, and antisense RNA silencing. First, epigenetic regulation is one process in which different classes of long and short RNAs cooperate to provide an astronomical number of functional outputs, some of which may drive cancer metastasis. As highlighted previously, many lncRNAs such as HOTAIR and H19 can recruit chromatin-modifying complexes to specific *loci*, thus silencing anti-metastatic genes and triggering metastatic transcriptional programs [39, 76]. In addition, there is also mounting evidence demonstrating that small regulatory RNAs can actively regulate chromatin dynamics by promoting gene repression through the RNAi-induced transcriptional silencing complex. Moreover, short RNAs called the piwi-interacting RNAs (piRNAs) are emerging as key players in epigenetic control [154]. Pioneering studies are beginning to show that at least some piRNAs are deregulated in cancer cells, and might play a role in metastatic progression, thereby adding a layer of complexity to the non-coding interactome [155]. Importantly, all these different NC-RNAs functionally synergize to critically regulate not only the expression of protein-coding genes but also the expression of all other NC-RNAs critical to cancer metastasis.

In addition to epigenetic regulation, alternative splicing represents the second molecular process in which a structurally diverse set of NC-RNAs interact to generate spatial and temporal complexity in cellular signaling pathways, underpinning phenotypic plasticity. Both snoRNAs and lncRNAs have been shown to co-localize in subnuclear compartments associated with RNA editing [143]. Additionally, many studies have highlighted their numerous functional contributions to splicing control, some of which are altered during tumor initiation and progression. Since many NC-RNAs function as part of ribonucleoprotein complexes, it seems likely that both snoRNAs and lncRNAs also associate with protein splicing factors, and that the resulting interaction underlies the splicing activity. We propose that the functional output of the splicing machinery will therefore be dictated by the NC-RNA composition (i.e., the non-coding transcriptome) of those regulatory complexes, which can vary over time in response to specific cues originating from the tumor microenvironment. Since some lncRNAs exhibit splice variant-dependent activity, splicing aberrations first initiated by deregulated NC-RNA expression have the potential to propagate further pro-metastatic changes by altering the activity of other lncRNAs. Similarly, the oncogenic capacity of many protein-coding genes is observed only in specific splice variants [156], which adds to the huge regulatory potential of the non-coding interactome underlying alternative splicing.

The third critical juncture in our proposed RNA-based molecular network involves the regulation of transcript stability by antisense RNAs. By post-transcriptionally regulating gene expression, this phenomenon has the potential to reprogram cellular phenotype rapidly in response to dynamic changes in the extracellular milieu. A highly diverse set of regulatory RNAs contribute to this process, consistent with the existence of an interconnected regulatory network controlled by NC-RNAs. Multiple RNA–RNA interactions are involved in this complex phenomenon, all of which can influence the functional output of this fundamental process. First, it has been extensively demonstrated that miRNAs play a central role by directly base pairing with target transcripts and inducing their degradation. A number of studies have highlighted the master regulatory role of miRNA networks in promoting cancer metastasis [144]. While providing a solid theoretical foundation for an RNA-based approach to cancer progression, we believe these studies do not fully account for all the complexity in this regulatory system. In fact, numerous recent studies have highlighted the importance of ceRNAs in this process, which act as miRNA buffers for protein-coding or other non-coding transcripts [105]. This critical role of ceRNAs implies that studies addressing differential miRNA expression need to consider the expression of ceRNA in interpreting the overall output of a miRNA-based pathway. Adding to the complexity of this aspect of the non-coding interactome, some auto-regulatory feedback loops between ceRNA and ceRNA-antisense transcripts (CATs) [157], as well as between short- and lncRNAs [153] have also been reported, and there are probably many more which have yet to be discovered. Finally, many miRNAs are directly derived from lncRNAs or snoRNAs, once again strengthening the hypothesis that an RNA-based molecular network controls phenotypic plasticity in human cancers.

The current classification of NC-RNAs is mainly based on transcript length and/or biogenesis, thereby ignoring the high degree of functional overlap shared by some of those molecules. As summarized in previous paragraphs, significant evidence indicates that NC-RNAs with different length may play very similar cellular functions, thereby suggesting the need for a classification system based not on transcript size but on molecular function. After reviewing the implications of a non-coding interactome underlying cancer metastasis, we now propose the first attempt to classify NC-RNA based on functionality. As mentioned before, epigenetic regulation involves a diverse spectrum of molecular interactions in which both short and long non-coding RNAs play master regulatory roles. To account for this functional overlap, we propose to group together all NC-RNAs known to be involved in controlling chromatin dynamics and name them “epigenetic co-factors”. At the same time, some lncRNAs and many snoRNAs share the property to interact with sRNP, thereby orchestrating mRNA splicing. This second class of splicing-related NC-RNAs could be named “non-coding splicing regulators”. Finally, it has been extensively demonstrated that small antisense transcripts generated by miRNA, snoRNA, and lncRNA *loci* can all be processed through Dicer-dependent mechanisms, leading to complementary RNA (either mRNA or NC-RNA silencing and degradation). We propose to collectively name this class of NC-RNAs “antisense-RNA-inhibitors”. This subclass will also include ceRNAs and CATs. The classification system we propose has the advantage of allowing one NC-RNA to be classified in many groups, which reflects the extensive functional diversity exhibited by the non-coding transcriptome. In addition, this system also allows the easy addition of novel subgroups as new molecular functions are discovered over time, making it flexible to the numerous paradigm changes that often arise in the search for scientific knowledge. In summary, the novel classification system we are proposing is based on function and not size, subclassifying individual NC-RNAs into three groups: (1) epigenetic co-factors, (2) non-coding splicing regulators, and (3) antisense RNA inhibitors. We believe that as research on NC-RNA proceeds, this classification system can become wider and more accurate.

It is worth noting that currently available NC-RNA databases mainly rely on bioinformatics algorithms for the identification of non-coding sequences [158]. Functional data confirming the non-coding nature of an RNA are limited to few exceptions [159]. With the notable exception of miRNAs, we currently lack a rational method for predicting NC-RNA interactions and targets. For example, most functional studies on lncRNA-polycomb interaction were triggered by co-expression analyses [160] or literature-based hypotheses [161]. A recently developed algorithm managed to predict up to 60 % of polycomb-binding lncRNAs in mouse cells [162], thereby paving the way to more comprehensive analyses in this field. Functional confirmation of non-coding function on a broader array of candidate NC-RNAs and a systematic approach to NC-RNA interaction discovery will likely enhance our understanding of the NC-interactome functions in pathological and physiological conditions.

Finally, we believe that the hypothesis of a NC-RNA interactome has clear implications for translational cancer research. It has been already proposed that NC-RNAs can be exploited as easy-to-detect biomarkers and therapeutic targets to block cancer progression [10, 144]. Properly engineered antisense oligonucleotides (ASOs) could be an effective tool to target pro-metastatic NC-RNAs. Some ASOs are currently being tested in phase III clinical trials, notably on patients with metastatic neoplasms [163]. Weekly administered ASOs presented an acceptable pharmacokinetic profile and were well tolerated by oncology patients [164, 165]. Most frequent grade 3 toxicities were fatigue and thrombocyto-lympho-penia. ASOs off-target effects include immuno-stimulation due to CpG-mediated Toll-like receptor activation [166]. This feature has raised some concerns on the possibility of inducing pro-survival effects in neoplastic cells as well. On the other hand, immunostimulatory effects could trigger an effective anti-cancer immune response [167], thereby potentiating ASO activity. It has been recently shown that targeted nanoparticle delivery can overcome ASO off-target effects and efficiently vehicle antisense drugs to metastatic lesions [166, 168]. NC-RNAs are also promising biomarkers. Indeed, one lncRNA (PCA3) is already used in the clinical setting as a biomarker for early prostate cancer detection [32]. Both snoRNAs and microRNAs can be detected in biological fluids from cancer patients [169], and effectively discriminate primary *vs*. metastatic disease [170]. A deeper understanding of the NC-RNA interactive network and its role in cancer metastasis might therefore translate into a wide range of clinical applications, ultimately bridging a fascinating research field with the urgent need for more effective cancer treatments.
